# Effects of Peanut Protein Supplementation on Resistance Training Adaptations in Younger Adults

**DOI:** 10.3390/nu13113981

**Published:** 2021-11-09

**Authors:** Casey L. Sexton, Morgan A. Smith, Kristen S. Smith, Shelby C. Osburn, Joshua S. Godwin, Bradley A. Ruple, Alex M. Hendricks, Christopher B. Mobley, Michael D. Goodlett, Andrew D. Frugé, Kaelin C. Young, Michael D. Roberts

**Affiliations:** 1 School of Kinesiology, Auburn University, Auburn, AL 36849, USA; cls0087@auburn.edu (C.L.S.); mas0216@auburn.edu (M.A.S.); sco0004@auburn.edu (S.C.O.); jsg0061@auburn.edu (J.S.G.); bar0049@auburn.edu (B.A.R.); amh0232@auburn.edu (A.M.H.); moblecb@auburn.edu (C.B.M.); kyoung@auburn.vcom.edu (K.C.Y.); 2 Department of Nutrition, Dietetics and Hospitality Management, Auburn University, Auburn, AL 36849, USA; kss0034@auburn.edu (K.S.S.); fruge@auburn.edu (A.D.F.); 3 Athletics Department, Auburn University, Auburn, AL 36849, USA; goodlmd@auburn.edu; 4 Department of Cell Biology and Physiology, Edward Via College of Osteopathic Medicine, Auburn, AL 36832, USA

**Keywords:** resistance training, females, peanut protein, muscle, protein synthesis

## Abstract

Protein supplementation is a commonly employed strategy to enhance resistance training adaptations. However, little research to date has examined if peanut protein supplementation is effective in this regard. Thus, we sought to determine if peanut protein supplementation (PP; 75 total g/d of powder providing 30 g/d protein, >9.2 g/d essential amino acids, ~315 kcal/d) affected resistance training adaptations in college-aged adults. Forty-seven college-aged adults (*n* = 34 females, *n* = 13 males) with minimal prior training experience were randomly assigned to a PP group (*n* = 18 females, *n* = 5 males) or a non-supplement group (CTL; *n* = 16 females, *n* = 8 males) (ClinicalTrials.gov trial registration NCT04707963; registered 13 January 2021). Body composition and strength variables were obtained prior to the intervention (PRE). Participants then completed 10 weeks of full-body resistance training (twice weekly) and PP participants consumed their supplement daily. POST measures were obtained 72 h following the last training bout and were identical to PRE testing measures. Muscle biopsies were also obtained at PRE, 24 h following the first exercise bout, and at POST. The first two biopsy time points were used to determine myofibrillar protein synthesis (MyoPS) rates in response to a naïve training bout with or without PP, and the PRE and POST biopsies were used to determine muscle fiber adaptations in females only. Dependent variables were analyzed in males and females separately using two-way (supplement × time) repeated measures ANOVAs, unless otherwise stated. The 24-h integrated MyoPS response to the first naïve training bout was similar between PP and CTL participants (dependent samples *t*-test *p* = 0.759 for females, *p* = 0.912 for males). For males, the only significant supplement × time interactions were for DXA-derived fat mass (interaction *p* = 0.034) and knee extensor peak torque (interaction *p* = 0.010); these variables significantly increased in the CTL group (*p* < 0.05), but not the PP group. For females, no significant supplement × time interactions existed, although interactions for whole body lean tissue mass (*p* = 0.088) and vastus lateralis thickness (*p* = 0.099) approached significance and magnitude increases in these characteristics favored the PP versus CTL group. In summary, this is the second study to determine the effects of PP supplementation on resistance training adaptations. While PP supplementation did not significantly enhance training adaptations, the aforementioned trends in females, the limited n-size in males, and this being the second PP supplementation study warrant more research to determine if different PP dosing strategies are more effective than the current approach.

## 1. Introduction

The recommended dietary allowance (RDA) for protein intake in adults is currently 0.8 g/kg body mass per day, but based on a rapidly expanding body of evidence, intakes around 1.2–1.6 g/kg of body mass per day are often recommended [1,2,3]. Higher protein intakes can be effective in increasing muscle mass and reducing body fat [1,2,4]. One strategy to increase protein intake is through supplementation. In recent years, protein supplementation has transitioned from being a niche practice in bodybuilding to a hallmark practice for both recreational and sports-related training [5].

Protein supplements derived from animal sources (i.e., whey, casein, egg albumin) are thought to be of higher quality than plant-based protein sources because the former protein sources often contain adequate amounts of all nine essential amino acids (EAAs) and have higher levels of leucine [6,7,8,9]. However, there has been a growing interest in the health benefits of plant-based foods as well as concerns related to the sustainability of procuring animal-based proteins [10]. Additionally, data from the National Health and Nutrition Examination Survey suggest that plant protein intake has increased significantly from 1999 to 2010 [11], and it has been posited that the increased intakes of plant-based food will continue to increase in decades to come [12]. With regard to efficacy, a meta-analysis from Lim et al. [13] compared how animal-based versus plant-based protein supplements affected resistance training adaptations in adults (<50 years old). The authors reported that animal protein only conferred modest improvements in lean body mass (0.41 kg) and percent lean mass (0.50%) compared to plant proteins. Likewise, Kerksick et al. [14] authored a comprehensive review suggesting plant protein-based supplements offering a sufficient amount of EAAs, especially leucine, can stimulate similar resistance training adaptations compared to animal protein sources. However, there are opposing data to suggest that animal-based protein supplements (e.g., whey protein) can elicit superior post-prandial anabolic responses in skeletal muscle versus soy, which is a complete plant-based protein [9]. This effect may be due to plant proteins, in general, containing less essential amino acids (on a g/100 g basis) as well as having limited amounts of certain essential amino acids (e.g., lysine and methionine) [15].

Protein derived from peanuts offers a full array of EAAs, only being low in methionine and threonine, and contains a protein digestibility-corrected amino acid score of 0.70/1.00 [16,17,18,19]. Although peanut protein is currently marketed with the intent of being a viable source of high-quality protein, it has been vastly understudied in the sports nutrition arena. In this regard, our laboratory has published the only study to date examining how peanut protein (PP) supplementation affected resistance training outcomes [18]. In short, we reported that older individuals (~60 years old) consuming ~30 g of PP on a daily basis better increased leg muscle hypertrophy and knee flexion torque compared to a control group of participants over 6–10 weeks of resistance training. However, younger participants were not examined. Therefore, the purpose of this study was to examine if 10 weeks of PP supplementation during resistance training affected body composition and indices of muscle size and strength in younger adults. Moreover, we sought to examine if post-exercise PP supplementation enhanced the myofibrillar protein synthetic response (MyoPS) over a 24-h period following the first naïve bout of resistance exercise in these same participants. Based on the prior data obtained in older individuals, we hypothesized that PP supplementation would increase various hypertrophy and strength characteristics compared to those that did not partake in supplementation.

## 2. Methods

### 2.1. Ethical Approval and Participant Inclusion Criteria

The Auburn University Institutional Review Board (IRB) reviewed and approved this study (Protocol # 19-249 MR 1907). Study procedures conformed to the latest standards established by the Declaration of Helsinki. The study was registered as a clinical trial prior to data collection (ClinicalTrials.gov trial registration NCT04707963; registered 13 January 2021). Participants were recruited from Auburn University’s campus and the surrounding area via email, flyers, and word of mouth. Eligible participants had to be male or female between the ages of 18–30 with a BMI < 35 kg/m^2^. Eligible participants could have also not resistance trained more than one time per week for six months prior to the study. Eligible participants also: (i) could not have a peanut allergy, (ii) had to be free of metal implants that may interfere with X-ray procedures, (iii) could not have obtained medically necessary radiation exposure (excluding dental X-rays) for six months prior, (iv) had to be free of obvious cardiovascular or metabolic disease, (v) had to be free of conditions contraindicating participation in exercise programs or donating muscle biopsies (i.e., taking blood thinners or blood clotting disorders), and (vi) for females, could not be pregnant or trying to become pregnant. Those that were deemed eligible and agreed to participate received an informed consent packet and were verbally informed of all study procedures. Following verbal and written consent, participants completed a health history questionnaire and scheduled a time for pre-testing (T1-1).

### 2.2. Study Design

**Figure 1:**
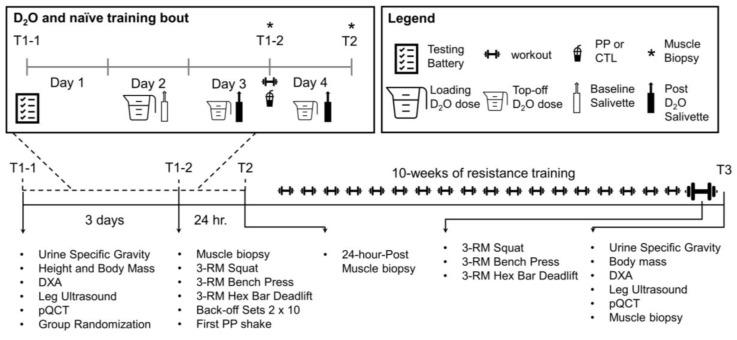
Study design. The figure above outlines the study design.

The timeline and testing battery for each visit can be visualized in Figure 1 and is described in greater detail below. Participants reported to the laboratory a total of 5 times for testing and 20 times for resistance training.

During the pre-testing visit (T1-1), participants reported to the laboratory at least four hours fasted and underwent an array of testing beginning with urine specific gravity (USG) to ensure hydration and a rapid pregnancy test for female participants. The following examinations were completed thereafter: (i) height and weight measurements, (ii) whole-body dual-energy X-ray absorptiometry (DXA), (iii) right leg mid-thigh peripheral quantitative computed tomography (pQCT) scan, (iv) vastus lateralis (VL) ultrasound of the mid-thigh, and (v) isokinetic dynamometry of the knee extensors. The participants also chewed a salivette (SARSTEDT AG & Co, Nümbrect, Germany) to provide saliva for the baseline assessment of whole-body deuterium oxide (D_2_O). At the end of T1-1, participants were given containers of D_2_O (70 atom percent; Sigma-Aldrich, St. Louis, MO, USA) to take home and consume over the next 3 days, a three-day food log, and were randomized to either the PP or the non-supplement control group (CTL). Regarding the self-administration of D_2_O, participants consumed a total of 4.5 mL per kg of lean/soft tissue mass prior to the first muscle biopsy.

The first biopsy/strength assessment visit (T1-2) took place three days after T1-1. During T1-2, participants chewed a salivette to monitor D_2_O enrichment, consumed a top-off dose of D_2_O (0.5 mL/kg), and donated skeletal muscle via biopsy prior to subsequently performing baseline strength testing on the bilateral leg press, barbell bench press, and hex-bar deadlift. This strength testing, with the addition of two sets of 10 repetitions on leg press, bench press, and deadlifts at 50% of their estimated 1-repetition maximum strength (1-RM), was considered the first/naïve training session. Thereafter, participants in the PP group consumed their first supplement dose mixed with ~16 fluid ounces of tap water (providing 75 total g/d of powder, 30 g/d protein, >9.2 g/d essential amino acids, ~315 kcal), and CTL participants did not consume a supplement. The PP supplement was PBfit (BetterBody Foods, Lindon, UT, USA) and the amino acid content per daily serving used in this study can be found in Table 1. Notably, our laboratory shipped this supplement to a third-party laboratory (Eurofins; Tucker, GA, USA) to determine total protein and total amino acid content [18].

Twenty-four hours after T1-2, participants returned to the laboratory at least four hours fasted for their second skeletal muscle biopsy visit (T2) and final salivette sampling for D_2_O enrichment. After T1-2, participants completed their 10-week resistance training program, where the last session included strength testing and isokinetic dynamometry. The last testing session (T3) was conducted at least 72 h following the last resistance training session was completed, and the following battery of tests were performed: (i) USG, body mass, (ii) DXA, (iii) pQCT, (iv) mid-thigh vastus lateralis ultrasound, and v) a muscle biopsy. Each of these tests is described in greater detail below.

### 2.3. Specific Testing Procedures

#### 2.3.1. Urine Specific Gravity

At the beginning of T1-1 and T3, participants donated a urine sample (~5 mL). The sample was immediately analyzed using a handheld refractometer (ATAGO; Bellevue, WA, USA) for urine-specific gravity (USG) levels. Participants with a USG value ≤ 1.030 were considered well hydrated. Those who exceeded a USG of 1.030 were excluded from the analysis.

#### 2.3.2. Body Composition

Following USG assessments, body mass and height were assessed using a laboratory scale (Seca 769; Hanover, MD, USA). Participants were then subjected to a whole-body dual-energy X-ray absorptiometry (DXA) scan (Lunar Prodigy; GE Corporation, Fairfield, CT, USA) to assess bone-free lean tissue mass (LTM) and fat mass (FM). This instrument was calibrated using a phantom device and quality-assurance was tested on each day that scans were performed. Prior to the test, participants were asked to remove any metallic objects and to lie supine on the DXA scanner table underneath the scanner arm. Following a 5-min period of allowing the participant to lay on the table, the scan was performed. Importantly, all scans were performed by the same investigator at both the pre- and post-testing time points. Test-retest reliability from our laboratory using intraclass correlation coefficient _3,1_ (ICC _3,1_), standard error of the measurement (SEM), and minimal difference to be considered real (MD) was previously determined for whole-body LTM on ten participants scanned approximately 24 h apart resulting in an ICC of 0.99, SEM of 0.36 kg, and MD of 0.99 kg.

#### 2.3.3. Peripheral Quantitative Computed Tomography

After undergoing the DXA scan, participants had a peripheral quantitative computed tomography (pQCT) scan (Stratec XCT 3000, Stratec Medical, Pforzheim, Germany) of the right thigh to measure whole muscle cross-sectional area (mCSA, cm^2^) and skeletal muscle density (mg/cm^3^) at 50% of the distance between the mid-inguinal crease and proximal patella. A permanent marker was used to indicate the precise transverse location of the scan and a mark was made mid-belly of the vastus lateralis so that subsequent ultrasound images and a muscle biopsy sample could be acquired from the exact location being imaged. Moreover, the biopsy scar was used as a reference point to ensure that post-testing (T3) images were captured at the same location as pre-testing. Each pQCT scan was captured using a scan speed of 20 mm/s, a 2.4 mm slice thickness, and a voxel size of 0.4 mm. Images were analyzed for total mCSA and muscle density using the pQCT BoneJ plugin [20] freely available through ImageJ analysis software (NIH, Bethesda, MD, USA). Importantly, all images were captured and analyzed by the same investigator who was blinded to group allocations. Test-retest reliability using ICC _3,1_, SEM, and MD was previously determined for mCSA on ten participants scanned approximately 24 h apart resulting in an ICC of 0.99, SEM of 0.84 cm^2^, and MD of 2.32 cm^2^.

#### 2.3.4. Ultrasound

Real-time B-mode ultrasonography (NextGen LOGIQe R8, GE Healthcare, Chicago, IL, USA) utilizing a multi-frequency linear-array transducer (L4-12T, 4–12 MHz, GE Healthcare, USA) was used to capture images of the vastus lateralis (VL) in the transverse plane for measurement of VL thickness (cm) and cross-sectional area (CSA, cm^2^). Prior to image acquisition, subjects rested supine on an examination table for a minimum of five minutes with the hip and knee fully extended. Images were captured at the same anatomical location as the pQCT scan as previously described above. For VL thickness, images were collected at a depth in which the edge of the femur was visible and this depth was held constant for post-testing image collection. For VL CSA measurements, a high-density cork pad was placed around the circumference of the thigh and secured using an adjustable strap. The pad was used as a guide for the consistent placement and movement of the probe in the transverse plane. All VL CSA images were captured using a panoramic function (LogicView, GE Healthcare, Chicago, IL, USA) with probe placement starting at the lateral aspect of the thigh and moving medially until the rectus femoris muscle was visible within the image. For all ultrasound images, a generous amount of water-soluble transmission gel was applied to both the skin and probe and care was taken to apply a consistent probe pressure to maximize image quality without compressing the underlying tissue. All ultrasound settings (frequency: 10 MHz, gain: 50 dB, dynamic range: 75), with the exception of depth, were held constant across participants and time points. One image per participant at each time point was obtained. Following the study conclusion, images were analyzed using the freely available ImageJ software (National Institutes of Health, Bethesda, MD, USA). VL thickness was measured using the straight-line function and defined as the distance between the subcutaneous adipose tissue–vastus lateralis interface and deep aponeurosis. VL CSA was calculated by manually tracing the border of the VL using the polygon function, with care taken to exclude any connective tissue within the region of interest. Again, these measurements were performed one time for each participant at each time point. All ultrasound images were captured and analyzed by the same investigator with a previously determined test-retest reliability in 10 participants using ICC_3,1_, SEM, and MD for VL thickness and VL CSA resulting in an ICC of 0.96 and 0.99, SEM of 0.09 cm and 0.60 cm^2^, and MD of 0.24 cm and 1.65 cm^2^, respectively.

#### 2.3.5. Isokinetic Dynamometry

Following ultrasound imaging, participants performed maximal right leg extensions on an isokinetic dynamometer (System 4 Pro, BioDex Medical Systems, Shirley, NY, USA). The seat of the dynamometer was adjusted so that the right knee was aligned with the axis of the dynamometer’s lever arm, the back of the knee had ~2–3 cm of clearance from the seat, and the hip was set to a 90° angle. Once positioned, participants were strapped to the chair so that the knee could only move in the sagittal plane about the frontal axis. The lever arm was fastened to the lower leg ~2–3 cm above the ankle. Maximal range of motion and limb weight were measured by the dynamometer. Peak extensor torque (60°/s) was then measured as the highest of five maximal extension attempts. During each set, participants were verbally encouraged to give maximal effort.

#### 2.3.6. Strength Testing

All participants began the testing session with a general warm-up consisting of 25 jumping jacks and 10 bodyweight squats, after which they completed a battery of strength testing in order of 45° leg press, flat barbell bench press, and hex-bar deadlifts. Each of the exercises began with 3–5 warm-up sets where load was incrementally increased in intensity based upon the participants’ perceived difficulty. Specifically, a load that was easily performed for 10 repetitions was first used, and this was followed by a load that could be completed for 5 repetitions. Testing finished with 1–3 sets of 3 repetitions with each of those sets increasing in intensity. As each set was completed, the participant was asked to rate the difficulty on a rating of perceived exertion (RPE) scale of 1–10 (“really easy” to “really hard”). An RPE of 1 resulted in a weight increase of 25%, an RP of 5 resulted in a 10% weight increase, and an RPE of 8–9 resulted in an incremental increase of 2–3%. Once a three-repetition maximum (3-RM) was achieved, this number was used to calculate an estimated 1-RM.

### 2.4. Skeletal Muscle Biopsies

Skeletal muscle biopsies were from the right leg vastus lateralis muscle collected at T1-2, T2, and T3. At T1-2, the biopsy was collected from the VL at the same location as the ultrasound and pQCT scans, and then at T2 and T3, the sample was taken 1–2 cm proximal of the initial sight. During biopsies, participants laid down on an athletic table where the upper thigh was shaven and cleansed with 70% isopropanol. A subcutaneous injection of 1% lidocaine (0.8 mL) was then administered. After 5 min, the area was cleansed with chlorhexidine solution. Thereafter, a pilot incision was made through the dermis with a sterile No. 11 surgical blade (AD Surgical; Sunnyvale, CA, USA). The 5-gauge biopsy needle was inserted into the pilot incision, through the muscle fascia, and ~2 cm into the muscle where a 50–100 mg sample was collected while applying suction [21]. Tissue was immediately removed from the needle, teased of blood and connective tissue, and separated for histological and tracer analysis. Tissue allocated for tracer analysis was placed in pre-labelled foils and immediately frozen at −80 °C via liquid nitrogen. Tissue allocated to histological analysis was embedded in optimum cutting temperature (OCT) gel to prevent freeze damage, slow-frozen in liquid nitrogen-cooled isopentane, and transported to −80 °C for storage until sectioned and stained.

### 2.5. Resistance Training Protocol and PP Supplementation

The training protocol was 10 weeks in duration and consisted of 20 separate training sessions (2 d/wk; Table 2). Each training session involved:

(i) A general warm-up of 25 jumping jacks and 10 body weight squats,
(ii) A specific warm-up of 1 set of 10 reps at 50% of working weight, 1 set of 5 repetitions at 75% of working weight, and 1 set of 3 repetitions of 90% of working weight,
(iii) Either 4 sets of 10 repetitions, or 5 sets of 6 reps per exercise

Regarding progressive overload, weekly loads increased by ~5% for the higher volume day and ~9% for the higher load day the first 4 weeks. Participants were given a week of reduced load training at week 5, which consisted of 50% intensity for both higher load and higher volume days. During week 6, progressive loading ensued from week 4 values. Although loads were pre-programmed for all participants, participant RPE was also used during each training session in order to ensure the appropriate load was implemented. Notably, participants in the PP group consumed their supplement after workouts under the supervision of staff, and CTL participants not consuming a supplement. Moreover, PP participants also consumed their supplement on non-workout days between meals but were instructed not to use the supplement as a meal replacement.

### 2.6. Wet Laboratory Analyses

*Immunohistochemistry.* T1-1 and T3 biopsies preserved in OCT were batch processed for: (i) cryostat sectioning, (ii) antibody staining, and (iii) imaging and analysis. Initially, all samples were sliced into 10 µm thick sections where they were electrostatically removed from the cooled cryostat (Leica Biosystems; Buffalo Grove, IL, USA) stage by a positively charged histology slide. The slides were then stored at −80 °C until all samples were ready to undergo antibody staining.

Staining for muscle fiber type (either type I or II) was performed as previously described by our laboratory [22]. Briefly, section-containing slides were removed from −80 °C storage and dried ~10 min at room temperature. Triton-X (0.5%) in phosphate buffer solution (PBS) was then used to permeabilize the sections for 5 min. This was followed by a 5-min wash in PBS and slides were subsequently incubated in a 100% concentration of blocking solution for 15 min (Pierce Super Blocker, Thermo Fisher Scientific, Waltham, MA, USA). The slides were then incubated in primary antibody solution for 60 min. This solution contained a 1× base of PBS, 5% of Pierce Super Blocker Solution, equal parts at 2% or a (1:50 dilution) of rabbit anti-dystrophin IgG1 (catalog #: GTX15277; Genetex Inc.; Irvine, CA, USA) and mouse anti-myosin I IgG1 (catalog #: A4.951 supernatant; Developmental Studies Hybridoma Bank, Iowa City, IA, USA). Slides were then washed for 5 min in PBS and then incubated in a secondary antibody solution containing a 1× base of PBS, equal parts at 1% or (1:100 dilution) Texas Red-conjugated anti-rabbit IgG (catalog #: TI-1000; Vector Laboratories, Burlingame, CA, USA) and Alexa Fluor 488-conjugated anti-mouse IgG1 (catalog #: A-11001; Thermo Fisher Scientific, Waltham, MA, USA). This incubation occurred in the dark for 60 min. The slides were then washed for 5 min in PBS, dried, and mounted using a 4,6-diamidino-2-phenylindole containing florescent media (DAPI; catalog #: GTX16206; Genetex Inc.). Immediately following mounting, slides were imaged using a fluorescent microscope (Nikon Instruments, Melville, NY, USA) with a 10× objective lens. Exposure times were 200 milliseconds for FITC, 600 milliseconds for TRITC, and 100 milliseconds for DAPI. Open-sourced software (MyoVision) was used to analyze all images for average fiber cross-sectional area (fCSA), muscle fiber type, and myonuclear number per fiber. A conversion of 0.964 μm/pixel was used to adjust the image for size and bit-depth, and a fiber size threshold was set at a minimum of 500 μm^2^ and a maximum of 15,000 μm^2^ to ensure the exclusion of spaces between fibers or fibers in an oblong orientation. Resultant images were also visually inspected for erroneous detection of fibers.

*Determination of MyoPS.* Saliva samples obtained in the laboratory (or returned by participants) were stored at −20 °C. Following the conclusion of the study, salivette tubes were centrifuged for 2 min at 1000× *g* (2 °C). Saliva was obtained thereafter and frozen at −20 °C. Frozen samples were shipped to Metabolic Solutions (Nashua, NH, USA) on dry ice for analysis. Saliva analysis for deuterium enrichment occurred using cavity ring-down spectroscopy. Instrumentation included Liquid Water Isotope Analyzer with an automated injection system and a version 2 upgrade (Los Gatos Research, Mountain View, CA, USA). Samples were vortexed and spun at 8000 rpm to remove any particulates. The aqueous phase of saliva was injected 6 times and the last three measurements were averaged for data analysis. Standard curves were generated before and after sample runs for the determination of deuterium enrichment. Intra-run precision using this method is generally <2 delta per mil (parts per thousand) and inter-run precision is generally <3.5 delta per mil.

Muscle biopsy samples from T1-1 and T1-2 were also batch-processed using our laboratory’s MIST method [23]. Isolated myofibrils were frozen at −80 °C and frozen samples were shipped on dry ice to Metabolic Solutions for tracer analyses. This process first involved hydrolyzing myofibril pellets for 18 h with 3 mL of 6 N HCl (100 °C). Dowex H^+^ resin (1 mL, 50 W × 8-100; Sigma-Aldrich, St. Louis, MO, USA) was also added to trap released alanine. Amino acid elution from the resin was performed using 2 mL of 3N NH_4_OH and eluates were evaporated to dryness. Thereafter, the N-acetyl, n-propyl (NAP) derivative of alanine was prepared, and the propyl ester was formed by adding 200 μL propyl acetate and 100 μL BF3:propanol (14%). Samples were heated at 110 °C for 30 min and solutions were evaporated to dryness under N_2_ gas at 60 °C. The N-acetyl group was formed by adding 100 μL of 0.1 M diethylamine (DEA) in hexane and 100 μL of acetic anhydride. This reaction was incubated for 20 min at 60 °C, and subsequently dried down with N_2_ gas and low heat. Samples were reconstituted in 100 μL ethyl acetate and pipetted into autosampler vials. Deuterated alanine from myofibrillar preparations was detected using a Thermo Finnigan Delta V IRMS coupled to a Thermo Trace GC Ultra with a GC combustion interface III and Conflow IV. The N-acetyl-n-propyl ester of alanine was analyzed using a splitless injection with CTC Pal autosampler (1 µL). Injections used a Zebron ZB-5 column of 30 m × 0.25 mm × 0.50 µm film thickness (Phenomenex, Torrance, CA, USA), and the injection temperature was 250 °C. The GC oven had an initial column temperature of 80 °C with a 2-min hold and this was followed by a ramp of 30 °C per minute to 330 °C. Compounds eluting off the column were directed into the pyrolysis reactor and were heated to 1450 °C hydrogen gas conversion. Deuterium enrichment was first expressed in delta values compared to a calibrated hydrogen gas. These values were then converted to atom % D by standard equations. Methylpalmitate was used as the calibration standard for the reference hydrogen gas. Intra-run precision for alanine measurements is generally <2 delta per mil, and inter-run precision is generally <3 delta per mil.

Saliva and myofibril enrichments were used to calculate MyoPS rates over the 24-h period following the first training bout. The equation follows that published by Bell et al. [24] (see equation below).

$$\mathrm{FSR}\;\left({\%\mathrm{day}}^{-1}\right)=\left[\frac{\left(E_{\mathrm{Ala}2}-E_{\mathrm{Ala}1}\right)}{E_{\mathrm{BW}}\times \;t}\right]\times 3.7\times 100$$

Briefly, the difference in deuterium (^2^H) enrichment from the first two biopsies (E_Ala2_ = T2 muscle sample enrichment, E_Ala2_ = T1 muscle sample enrichment) is divided by the product total body enrichment of ^2^H (in atom % excess) (E_BW_ = ^2^H from T1-2 and T2 saliva − Baseline ^2^H from T1-1saliva) and number of days that D_2_O was consumed at the loading dose. This quotient is then multiplied by 3.7 to adjust for the number of ^2^H atoms typically bound to alanine. Finally, the resultant value is multiplied by 100 to achieve myofibrillar synthesis rate in percent per day.

### 2.7. Food Log Analysis

The three-day food log packets requested nutritional intakes for one weekend day and two weekdays in the week leading up to the pre-intervention (T1-2) and last testing session (T3). Participants were asked to maintain their normal dietary habits through the duration of the study, except daily consumption of the protein supplement in the PP group. Data from the food logs were entered into the Nutrition Data System for Research (NDSR) (NDSR 2014: University of Minnesota). Calories, nutrients, and food groups were averaged from the three days of food logs for a mean intake (g/d or g/kg/d) at each time point.

## 3. Statistical Analysis

Statistical analysis was performed in SPSS v26.0 (IBM Corp, Armonk, NY, USA). Prior to statistical analysis, normality testing was performed on all dependent variables using Shapiro–Wilk tests at the T1 and T3 time points within each gender. The only data that were non-normally distributed were T3 1RM bench press and T3 1RM deadlift values in PP females (*p* = 0.035 and *p* = 0.049, respectively). Given that an overwhelming majority of the data were normally distributed, we proceeded to analyze data using parametric statistical tests.

Independent samples *t*-tests were used to compare the 24-h MyoPS rates across groups. Two-way (supplement × time) repeated measure ANOVAs were used to determine changes in dependent variables over time. Statistical significance was established at *p* < 0.05. When a significant supplement × time interaction was observed, LSD post hocs were performed to determine differences within each group from pre- to post-intervention and between groups at each time point. For select variables, Cohen’s d effects sizes were also calculated within groups by obtaining the mean difference score for the variable of interest and dividing it by the pooled standard deviation of said variable. In line with a recent publication from our laboratory, effect sizes were classified as small (d < 0.2), medium (d > 0.21, d < 0.5), or large (d > 0.51) [25]. For all statistical analyses, males and females were analyzed separately.

## 4. Results

### 4.1. Consort Diagram

**Figure 2:**
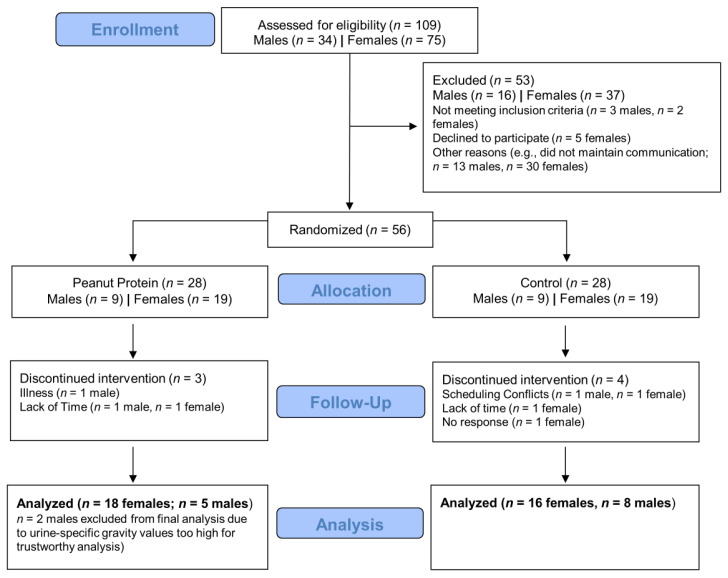
CONSORT diagram. The diagram indicates how many individuals were screened and completed the intervention.

Figure 2 provides a detailed CONSORT diagram of the study. Briefly, 109 potential participants contacted the study coordinator. Of these, 56 were eligible and agreed to participate in the study and *n* = 28 were randomized to the PP group whereas *n* = 28 were randomized to the CTL group. Three participants in the PP group had to discontinue the study due to an illness (*n* = 1) or lack of time (*n* = 2), and four CTL participants discontinued the study for reasons detailed in the figure. Notably, more females completed the study relative to males (PP, males = 7 and females = 18; CTL, males = 8 and females = 16). At the end of the study, it was also discovered that two males in the PP group yielded T3 USG values that were above 1.030, and these males were excluded. Thus, this yielded a final male count of PP *n* = 5 and CTL *n* = 8.

### 4.2. Baseline Participant Characteristics

Baseline participant characteristics between the PP and CTL cohorts are presented in Table 3. There were no differences between supplementation groups regarding age, height, or body composition characteristics for either gender.

### 4.3. MyoPS Response to First Bout of Training

For this analysis, 34 female participants had sufficient salivette enrichment (PP *n* = 18, CTL *n* = 16), with 32 of those participants (PP *n* = 18, CTL *n* = 14) having appropriate yields from isolated myofibrillar pellets. Thus, two female CTL participants were not included in this analysis. For males, 12 participants had sufficient salivette enrichment (PP *n* = 5, CTL *n* = 7), and all of these participants provided appropriate yields from isolated myofibrillar pellets. Thus, one male CTL participant was not included.

**Figure 3:**
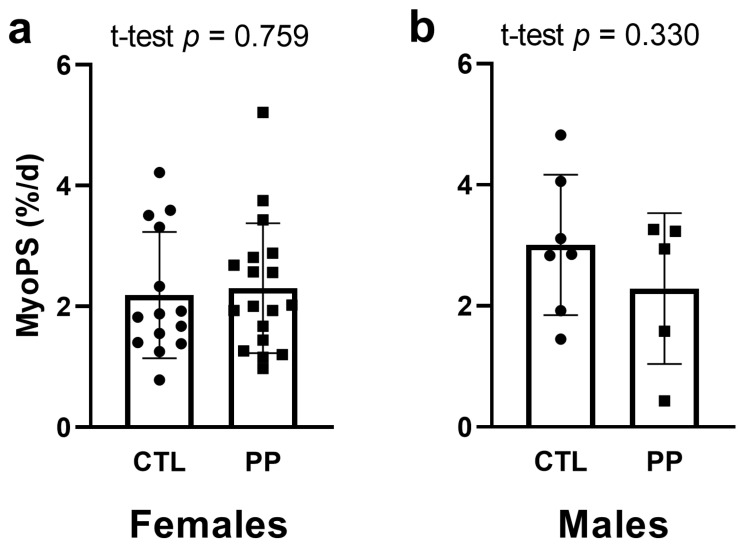
Myofibrillar protein synthesis rates following the first bout of training with or without PP supplementation. Data include myofibrillar protein synthesis rates 24 h following the first exercise bout in females (panel **a**) and males (panel **b**). All data are presented as mean ± standard deviation values, and individual respondent values are presented as circles (for CTL) and squares (for PP). Abbreviations: PP, peanut protein group; CTL, control group.

For females, no significant difference existed for the 24-h integrated MyoPS response 24 h following the first bout of training between PP and CTL groups (*p* = 0.759) (Figure 3a). Similarly, no significant difference existed for the 24-h integrated MyoPS response 24 h following the first bout of training between PP and CTL male participants (*p* = 0.330) (Figure 3b).

### 4.4. Nutritional Intakes Prior to and at the End of the 10-Week Intervention

All 34 female participants (*n* = 18 PP, *n* = 16 CTL) provided viable 3-day food logs for both T1 and T3 (Table 4). With the inclusion of the PP supplement, there was no significant interactions for daily energy (*p* = 0.652), carbohydrate (*p* = 0.096), or fat intakes (*p* = 0.723). There was a significant interaction for daily protein intake (*p* = 0.006) and relative protein intake (g/kg) (*p* = 0.008). Post hoc analysis indicated that these values increased in the PP group from pre- to post-intervention. Additionally, T3 values were greater in the PP versus CTL group.

For males, 12 participants (*n* = 4 PP, *n* = 8 CTL) provided viable 3-day food logs for both T1 and T3 (Table 4). With the inclusion of the PP supplement, there was no significant interactions for daily energy (*p* = 0.391), carbohydrate (*p* = 0.209), fat (*p* = 0.800), or protein intakes (*p* = 0.775).

### 4.5. Body Composition Changes with Training in PP versus CTL

**Figure 4:**
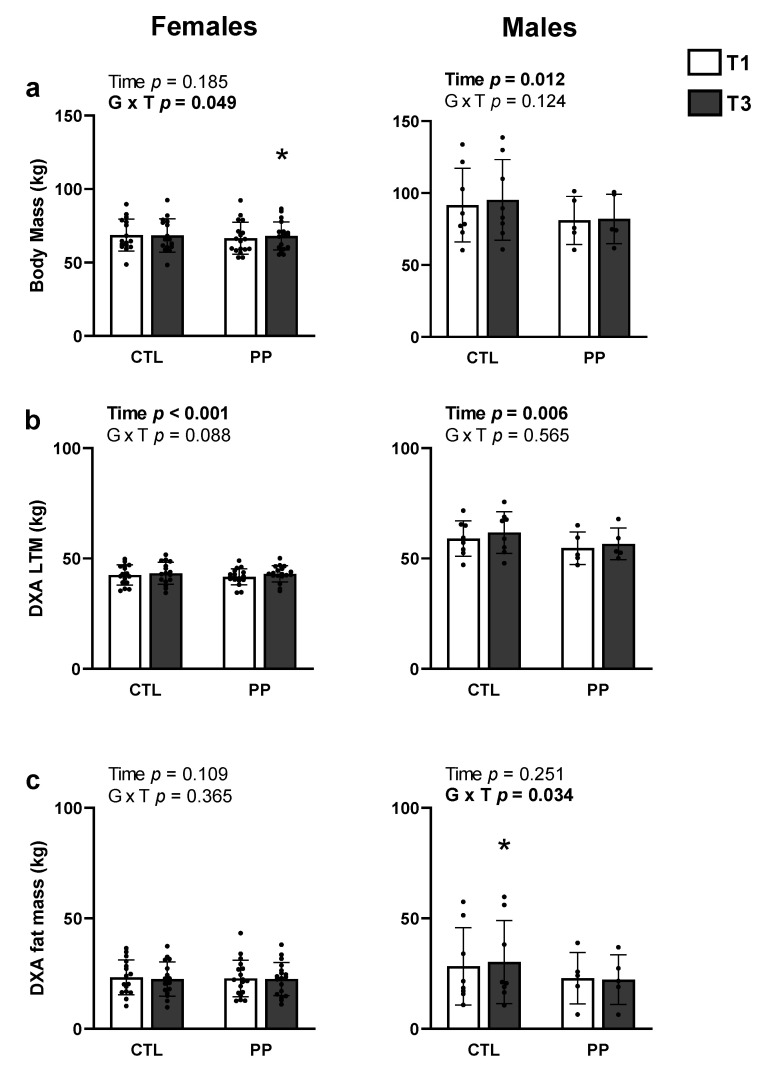
Body composition adaptations. Data include body mass (**a**), DXA-derived lean/soft tissue mass (**b**), and DXA-derived fat mass (**c**). All data are presented as mean ± standard deviation values. Abbreviations: PP, peanut protein group; CTL, control group. Symbols: •, indicates individual respondent data; *, indicates increase (*p* < 0.05) within supplementation group from pre- to post-intervention.

For females, there were significant supplement × time interactions for body mass (*p* = 0.049) (Figure 4a). Post hoc analyses indicated values increased in the PP group from pre to post-intervention (*p* < 0.05), but not the CTL group. No significant supplement × time interactions existed for DXA LTM (*p* = 0.088; Figure 4b), or DXA fat mass (Figure 4c).

For males, a significant supplement × time interaction existed for DXA fat mass (Figure 4c). Post hoc analyses indicated these values increased in the CTL group from pre to post-intervention (*p* < 0.05), but not the PP group. No significant supplement × time interactions existed for body mass (Figure 4a) or DXA LTM (Figure 4b).

### 4.6. Mid-Thigh Characteristics with Training in PP versus CTL

**Figure 5:**
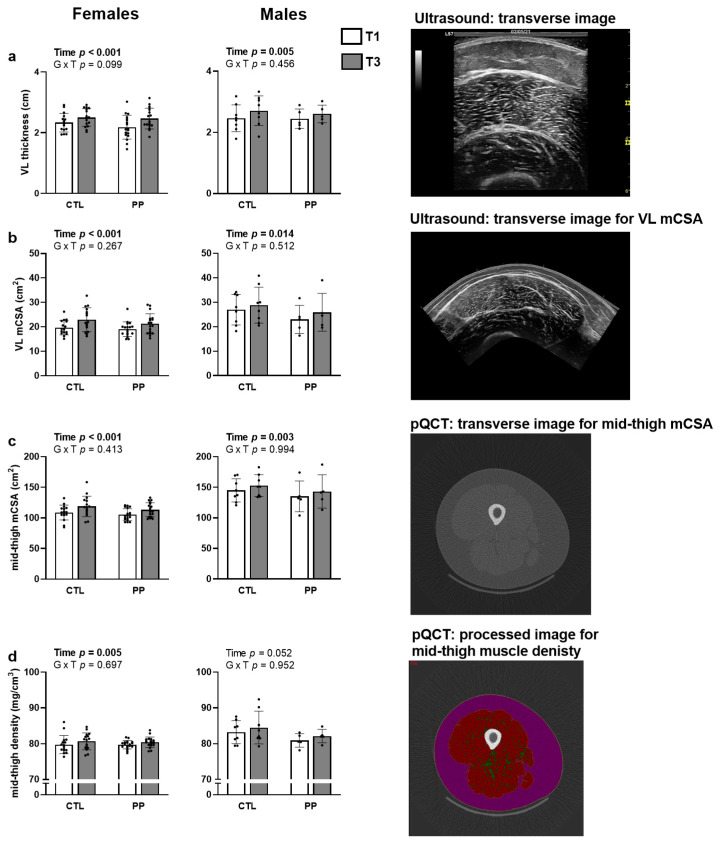
Mid-thigh muscle adaptations. Data include vastus lateralis (VL) thickness (**a**), vastus lateralis muscle cross-sectional area (mCSA; (**b**)), mid-thigh mCSA (**c**), and mid-thigh muscle density (**d**). Representative images of each technique are provided to the right of each bar graph. All data are presented as mean ± standard deviation values. Other abbreviations: PP, peanut protein group; CTL, control group. Symbol: •, indicates individual respondent data.

For females, a supplement × time interaction trended for vastus lateralis thickness (*p* = 0.099; Figure 5a), but no significant interaction effects existed for vastus lateralis mCSA (*p* = 0.267; Figure 5b), mid-thigh mCSA (*p* = 0.413; Figure 5c), or mid-thigh muscle density (*p* = 0.697; Figure 5d).

For males, no significant supplement × time interactions existed for vastus lateralis thickness (*p* = 0.456; Figure 5a), vastus lateralis mCSA (*p* = 0.512; Figure 5b), mid-thigh mCSA (*p* = 0.994; Figure 5c), or mid-thigh muscle density (*p* = 0.952; Figure 5d).

### 4.7. Total Training Volume and Strength Adaptations with Training in PP versus CTL

Total training volume and strength adaptations are presented in Table 5. For females, estimated 1-RM values for leg press, bench press, and deadlift all increased over time (*p* < 0.001 for all variables), but no significant interactions existed for these variables (*p* = 0.468, 0.223, and 0.350, respectively). Isokinetic knee extensor peak torque did not exhibit a significant time effect (*p* = 0.075) or interaction (*p* = 0.449), and total training volume did not differ between supplementation groups (*p* = 0.577).

For males, estimated 1-RM values leg press, bench press, and deadlift all also increased over time (*p* < 0.001 for all variables), but no significant interactions existed for these variables (*p* = 0.479, 0.400, and 0.084, respectively). Isokinetic knee extensor peak torque did exhibit a significant time effect (*p* = 0.023) and significant interaction (*p* = 0.010). Post hoc analysis indicated that this variable increased in the CTL group (*p* = 0.005), but not the PP group (*p* = 0.688). Total training volume did not differ between supplementation groups (*p* = 0.698).

### 4.8. Muscle Fiber Adaptations with Training in PP versus CTL in Female Participants

Due to limited n-sizes, male participants were not analyzed for muscle fiber adaptations. For females, no significant interactions existed for type I fCSA (*p* = 0.410), type I fiber myonuclear number (*p* = 0.564), type II fCSA (*p* = 0.126), type I fiber myonuclear number (*p* = 0.619).

## 5. Discussion

This is the second study to examine how PP supplementation affects resistance training adaptations and is the first to provide these data in younger adult females. In short, the only variables to demonstrate significant interaction effects included body mass and BMI, and both variables significantly increased in the PP group. The interaction for whole-body DXA LTM approached significance in females (*p* = 0.088) and PP participants demonstrated greater increases in this variable (1.4 ± 1.2 kg) versus CTL (0.7 ± 0.8 kg). Additionally, the calculated effect size was moderate for the PP group (d = 0.378) and small for the CTL group (d = 0.151). Likewise, the interaction for vastus lateralis thickness also approached significance (*p* = 0.099) in females and PP participants demonstrated greater increases in this variable (0.28 ± 0.20 cm) versus CTL participants (0.17 ± 0.18 cm). Additionally, the calculated effect size was larger for the PP group (d = 0.772) compared to the CTL group (d = 0.576). However, other mid-thigh variables (i.e., muscle fiber CSAs in females and mid-thigh CSA values in both genders) also did not demonstrate significant interactions. Thus, the implemented PP supplementation strategy (i.e., 30 g/d) was largely ineffective at promoting superior training adaptations relative to no supplementation.

We posit that several of the null findings herein are due to either: (i) this population’s naïve training response being too robust to be influenced by nutritional factors, or (ii) the protein dose being provided by PP supplementation not being large enough to elicit further adaptation over a 10-week period. The former contention is supported by a recent meta-analysis from Morton and colleagues showing that protein supplementation was more effective in increasing muscle mass in persons that were previously trained [3], whereas our cohort was untrained. Moreover, the same meta-analysis suggests that protein intakes equal to or greater than 1.62 g/kg/day optimizes increases in lean body mass during resistance training. Indeed, it is difficult to reconcile why a similar PP supplementation regimen increased vastus lateralis hypertrophy and knee extensor strength in older participants during 6–10 weeks of resistance training but did not show significant effects herein. However, in line with the second point above, older participants in our prior study who supplemented with PP increased their daily protein intakes from ~1.2 g/kg/d to ~1.5 g/kg/d (*p* < 0.05). In the current study, PP supplementation in females increased daily intakes from ~1.0 g/kg/d to ~1.4 g/kg/d (*p* < 0.05). While the data were limited to *n* = 4 younger males who completed food logs in the current study, PP supplementation did not significantly alter self-reported intakes in this cohort (PRE = 1.6 ± 0.8 g/kg/d to 1.7 g/kg/d, *p* = 0.853). Hence, older persons supplementing with PP seemingly increased their daily protein intake to a threshold that stimulated anabolic effects, whereas younger females in the current study did not. Additionally, given that PP supplementation minimally influenced daily protein intakes in males, this may have been responsible for the null anabolic findings. In lieu of the collective data, more protein through PP supplementation may have been needed to elicit further gains in hypertrophy in younger participants and future studies implementing higher PP doses (e.g., two doses per day providing a total of 60 g supplemental protein) are warranted.

PP supplementation after one bout of resistance exercise also did not enhance integrated MyoPS rates within a 24-h period following the first training bout. This is the second time that we have observed this given that our prior study in older participants yielded similar outcomes [18]. In our prior study, we posited that had we sampled muscle in closer proximity following exercise, we may have observed enhanced post-exercise MyoPS rates with PP compared to no supplementation. We maintain this hypothesis given that several studies have shown protein ingestion following resistance training elicits significant increases in muscle protein synthesis or MyoPS within a 3–12 h window under controlled laboratory conditions [9,26,27,28]. However, this issue remains unresolved and warrants future research.

In the context of the current findings and given that we did not examine a third group of participants consuming an animal-sourced protein supplement (e.g., whey), it is difficult to determine how PP supplementation compares to whey protein supplementation. Additionally, this being the second PP study further confounds this comparison. However, other studies have examined acute and chronic effects of ingesting soy versus whey protein on anabolic outcomes. For instance, Tang et al. [9] determined in college-aged participants that the consumption of soy protein did not optimize post-exercise increases in muscle protein synthesis compared to an isonitrogenous dose of whey protein. Volek et al. [29] determined that whey protein supplementation (21.6 g/d) in college-aged participants over a 9-month period significantly increased whole-body lean mass compared to soy protein supplementation (20 g/d). The authors of both studies suggest the superior essential amino acid profile of whey versus soy protein was likely responsible for the observations. Essential amino acids make up ~50% whey protein and ~36% soy protein [30]. Essential amino acids make up ~30% PP according to recent third-party testing performed on the PP supplement [18]. Thus, PP supplementation likely elicits physiological effects that are more similar to soy versus whey protein. However, since the data are limited on PP supplementation, comparative studies are warranted.

Finally, there were interesting interactions worthy of discussion in the male cohort. Specifically, whole-body fat mass increased in the CTL group, whereas this increase did not occur in the PP group. In addition, knee extensor peak torque increased in the CTL group, whereas this variable did not change in the PP cohort. With regard to the former finding, higher protein intakes have been shown to stimulate a loss in body fat in those that resistance train [31]. However, this is difficult to reconcile given that PP supplementation did not affect daily protein intakes in the male cohort. There is no plausible explanation as to why knee extensor peak torque increased in the CTL group and did not in the PP group. It is unlikely that PP supplementation is ergolytic given that the vast majority of the literature suggests that increased protein intake, whether it be from plant- or animal-based foods, elicits anabolic effects. Notwithstanding, both of these findings in males are highly confounded with the n-size limitations. Thus, more research is needed to replicate both findings prior to speculating potential mechanisms of action.

### Limitations

A 10-week period is limited in scope and duration and longer-term interventions are warranted. Moreover, the low recruitment of males as well as the attrition of male participants provides an incomplete analysis in this gender. This is an unresolved limitation and, given an overall lack of data in relation to PP supplementation, warrants future studies with this population. Finally, we currently lack data regarding how 30 g of PP consumption, or PP consumption in general, affects postprandial levels of essential amino acids. Indeed, these are critical data to consider given that the ability of a dietary protein source to increase essential amino acids is related to its anabolic potential [9].

## 6. Conclusions

In conclusion, this is the second study to determine the effects of PP supplementation on resistance training adaptations. Given the aforementioned statistical trends, more research is needed to determine if greater PP dosing or longer training periods with PP supplementation is more effective than the current approach.

## Figures and Tables

**Table 1:** Amino acid content in PP per daily serving.

Variable	Amount Per Daily Serving (3 Scoops or 75 g Powder)
Total protein ^1^	30.1 g
Essential ^2^	
Histidine	0.72 g
Isoleucine	1.14 g
Leucine	2.17 g
Lysine	0.87 g
Methionine	0.41 g
Phenylalanine	1.66 g
Threonine	0.86 g
Tryptophan	ND
Valine	1.37 g
Total essential	9.2 g
Non-essential	
Alanine	1.37 g
Arginine	3.71 g
Asparagine	3.77 g
Cysteine	0.45 g
Glutamic acid	6.26 g
Glycine	1.90 g
Proline	1.37 g
Serine	1.49 g
Tyrosine	1.35 g
Total non-essential	21.7 g

Legend: These data represent the amino acid profile of the PP supplement. Notes: ^1^, these data were obtained using the Dumas method; ^2^, these data were obtained according to Eurofin’s Agilent Application note 5990-4547 (2010); ND, not determined.

**Table 2:** Participant baseline characteristics.

Variable	PP	CTL	*p*-Value
Females			
Age (years)	22 ± 2	21 ± 1	0.611
Height (cm)	171 ± 5	169 ± 5	0.176
Body mass (kg)	66.6 ± 10.8	68.7 ± 10.9	0.565
DXA FM (kg)	22.8 ± 8.3	23.2 ± 7.9	0.863
DXA LTM (kg)	41.7 ± 3.6	42.6 ± 4.6	0.529
VL thickness (cm)	2.18 ± 0.39	2.33 ± 0.30	0.229
Males			
Age (years)	21 ± 2	21 ± 1	0.304
Height (cm)	177 ± 7	180 ± 8	0.506
Body mass (kg)	76.0 ± 14.2	91.6 ± 25.5	0.431
DXA FM (kg)	18.9 ± 8.7	28.3 ± 17.5	0.555
DXA LTM (kg)	53.5 ± 7.9	61.8 ± 9.5	0.347
VL thickness (cm)	2.39 ± 0.34	2.71 ± 0.49	0.948

Legend: Data are presented as means ± standard deviation values for females (PP = 18, CTL = 16) and males (PP = 5, CTL = 8). Abbreviations: PP, peanut protein group; CTL, control group; cm, centimeters; kg, kilograms; DXA FM, fat mass determined by dual-energy X-ray absorptiometry; DXA LTM, lean tissue mass determined by dual-energy X-ray absorptiometry.

**Table 3:** Self-reported food log data.

Variable	Group	T1	T3 (Includes PP)
Females			
Energy intake (kcal/d)	PP CTL	1495 ± 430 1419 ± 458	1607 ± 513 1359 ± 327
Fat intake (g/d)	PP CTL	68 ± 19 59 ± 18	66 ± 25 60 ± 16
Carbohydrate intake (g/d)	PP CTL	152 ± 61 163 ± 83	167 ± 65 151 ± 45
Protein intake (g/d)	PP CTL	69 ± 29 ^a^ 60 ± 25	91 ± 23 ^b,c^ 51 ± 15
Protein intake (g/kg body mass/d)	PP CTL	1.02 ± 0.40 0.87 ± 0.32	1.36 ± 0.37 ^b,c^ 0.87 ± 0.27
Males			
Energy intake (kcal/d)	PP CTL	2262 ± 766 1727 ± 656	2433 ± 685 1298 ± 324
Fat intake (g/d)	PP CTL	110 ± 29 83 ± 44	100 ± 32 53 ± 16
Carbohydrate intake (g/d)	PP CTL	197 ± 108 156 ± 80	245 ± 70 130 ± 37
Protein intake (g/d)	PP CTL	118 ± 38 90 ± 41	128 ± 17 74 ± 22
Protein intake (g/kg body mass/d)	PP CTL	1.62 ± 0.83 1.06 ± 0.53	1.68 ± 0.36 0.87 ± 0.43

Legend: Self-reported intakes are presented as means ± standard deviation values (females: PP *n* = 18, CTL *n* = 16; males: PP *n* = 4, CTL *n* = 8). This table represents values at T1 and T3 with the addition of a PP supplement in the PP group. Abbreviations: PP, peanut protein group; CTL, control group; g, grams; kg, kilograms; kcal, kilocalories. Symbols for females: ^a^, denotes PP is significantly different from CTL at T1; ^b^, denotes a significant increase within PP from T1 to T3; ^c^, denotes PP is significantly different from CTL at T3.

**Table 4:** Resistance exercise training program.

Week	Day	Sets × Repetitions	%1RM
1	1	3-RM Testing (+2 × 10)	50%
	2	5 × 6	56%
2	3	4 × 10	55%
	4	5 × 6	65%
3	5	4 × 10	60%
	6	5 × 6	74%
4	7	4 × 10	65%
	8	5 × 6	84%
5	9	4 × 10	50%
	10	5 × 6	50%
6	11	4 × 10	65%
	12	5 × 6	84%
7	13	4 × 10	70%
	14	5 × 6	90%
8	15	4 × 10	75%
	16	5 × 6	96%
9	17	4 × 10	80%
	18	5 × 6	98%
10	19	5 × 6	102%
	20	3-RM Testing	---

Legend: This table represents the training paradigm for the 10-week progressive resistance training program. Abbreviations: 3-RM, three-repetition maximum test.

**Table 5:** Strength adaptation data.

Variable	Group	T1	T3
Females			
Leg press 1RM (kg)	PP CTL	79 ± 39 88 ± 39	160 ± 41 177 ± 55
Bench press 1RM (kg)	PP CTL	31 ± 6 32 ± 7	38 ± 6 41 ± 8
Deadlift 1RM (kg)	PP CTL	59 ± 14 62 ± 15	82 ± 19 88 ± 18
Leg extensor peak torque (N × m)	PP CTL	129 ± 32 141 ± 30	135 ± 38 155 ± 33
Total training volume (kg)	PP CTL	131,728 ± 27,867 137,228 ± 29,098	
Males			
Leg press 1RM (kg)	PP CTL	152 ± 41 214 ± 53	260 ± 105 366 ± 87
Bench press 1RM (kg)	PP CTL	69 ± 19 74 ± 20	80 ± 19 83 ± 19
Deadlift 1RM (kg)	PP CTL	102 ± 31 111 ± 16	125 ± 38 151 ± 23
Leg extensor peak torque (N × m)	PP CTL	174 ± 30 184 ± 36	171 ± 37 225 ± 42 *
Total training volume (kg)	PP CTL	280,345 ± 116,800 312,335 ± 152,903	

Legend: Self-reported intakes are presented as means ± standard deviation values (females: PP *n* = 18, CTL *n* = 16; males: PP *n* = 4, CTL *n* = 8). This table represents values at T1 and T3 with the addition of a PP supplement in the PP group. Abbreviations: 1RM, estimated one-repetition maximum from a 3-RM assessment; kg, kilograms; N × m, Newton×meters. Symbols for males: *, denotes a significant increase within CTL from T1 to T3.

## Data Availability

All raw data can be obtained by emailing the corresponding author (mdr0024@auburn.edu).
